# The effect of an anti-malarial subsidy programme on the quality of service provision of artemisinin-based combination therapy in Kenya: a cluster-randomized, controlled trial

**DOI:** 10.1186/1475-2875-12-81

**Published:** 2013-03-01

**Authors:** Beth P Kangwana, Sarah V Kedenge, Abdisalan M Noor, Victor A Alegana, Andrew J Nyandigisi, Jayesh Pandit, Greg W Fegan, Jim E Todd, Robert W Snow, Catherine A Goodman

**Affiliations:** 1 Malaria Public Health Department, Kenya Medical Research Institute-Wellcome Trust Research Programme, Nairobi, Kenya; 2 Centre for Tropical Medicine, Nuffield Department of Clinical Medicine, University of Oxford, CCVTM, Oxford, UK; 3 Division of Malaria Control, Ministry of Public Health and Sanitation, Nairobi, Kenya; 4 Pharmacy and Poisons Board, Nairobi, Kenya; 5 London School of Hygiene and Tropical Medicine, London, UK

**Keywords:** Community case-management of malaria, Artemisinin-based combination therapy, Antimalarial subsidy programme, Drug retailers

## Abstract

**Background:**

Many patients with suspected malaria in sub-Saharan Africa seek treatment from private providers, but this sector suffers from sub-standard medicine dispensing practices. To improve the quality of care received for presumptive malaria from the highly accessed private retail sector in western Kenya, subsidized pre-packaged artemether-lumefantrine (AL) was provided to private retailers, together with a one day training for retail staff on malaria diagnosis and treatment, job aids and community engagement activities.

**Methods:**

The intervention was assessed using a cluster-randomized, controlled design. Provider and mystery-shopper cross-sectional surveys were conducted at baseline and eight months post-intervention to assess provider practices. Data were analysed based on cluster-level summaries, comparing control and intervention arms.

**Results:**

On average, 564 retail outlets were interviewed per year. At follow-up, 43% of respondents reported that at least one staff member had attended the training in the intervention arm. The intervention significantly increased the percentage of providers knowing the first line treatment for uncomplicated malaria by 24.2% points (confidence interval (CI): 14.8%, 33.6%; adjusted p=0.0001); the percentage of outlets stocking AL by 31.7% points (CI: 22.0%, 41.3%; adjusted p=0.0001); and the percentage of providers prescribing AL for presumptive malaria by 23.6% points (CI: 18.7%, 28.6%; adjusted p=0.0001). Generally outlets that received training and job aids performed better than those receiving one or none of these intervention components.

**Conclusion:**

Overall, subsidizing ACT and retailer training can significantly increase the percentage of outlets stocking and selling AL for the presumptive treatment of malaria, but further research is needed on strategies to improve the provision of counselling advice to retail customers.

## Background

Artemisinin-based combination therapy (ACT) has been incorporated into national policies for the treatment of uncomplicated malaria in all *Plasmodium falciparum*-endemic countries in Africa [1,2]. Many patients with suspected malaria in sub-Saharan Africa seek treatment from private providers [3-5], but this sector has been associated with substandard practices, such as misdiagnoses, poor counselling and incorrect dosing [4,6]. In addition, private sector retail prices for ACT have been high compared to those for less effective, but more widely used monotherapy, such as amodiaquine or sulphadoxine-pyrimethamine (SP) [7]. Barriers in the private and public sectors to accessing ACT treatment have contributed to the finding that less than 22% of children with fever were treated with an ACT across six African countries [8]. It is, therefore, important to investigate ways to improve the quality of malaria case-management in the private sector. Attention has focused on a range of strategies, including shopkeeper training, pre-packaging of drugs, community education, and most recently, ACT subsidies [9-11].

In Kenya, previous pilot studies have shown that interventions that included training of general shopkeepers could improve malaria case-management [12-15]. However, these interventions were carried out when anti-malarial monotherapies such as SP and amodiaquine were still effective and were the first line treatment for uncomplicated malaria. The introduction of AL as first line treatment posed major challenges to the shopkeeper training strategies since AL was less well-known than the monotherapies, and required specific knowledge and dispensing practices. Existing policies to train shopkeepers on older monotherapies were no longer appropriate or likely to have a significant impact on coverage of appropriate treatment. Here data are presented from provider and mystery-shopper surveys conducted in western Kenya, as part of a cluster-randomized, controlled trial of an ACT subsidy programme, which included shopkeeper training and community awareness activities, to evaluate its impact on private sector ACT availability, provider knowledge, and provider dispensing practices.

## Methods

### Study sites

The study was conducted in three districts in western Kenya (Busia, Butere-Mumias and Teso) where the first-line treatment for uncomplicated malaria was the ACT artemether-lumefantrine (AL). These areas were selected because of the presence of a relatively active retail market, and their high malaria endemicity [16]. Across the three districts, the percentage of the population living below the poverty line averaged 60% [17]. At the time of the study, Butere-Mumias had 51 government health facilities, Busia 39 and Teso 21, consisting of dispensaries, health centres and one district hospital per district (Noor, unpublished observations). All government health facilities in Kenya are supposed to supply AL free to patients, although stock-outs and unofficial fees are common [18,19]. The three main types of retail outlets supplying anti-malarials in Kenya were registered and unregistered pharmacies (together termed specialized drug stores), and general stores. Registered pharmacies are generally rare in rural areas. Artemether-lumefantrine was a prescription-only medicine, officially available at registered health facilities and pharmacies only, although in practice many prescription-only drugs were dispensed without a prescription in pharmacies and other retail outlets. At the time of the study, AL had a retail price of around 6.16 US dollars (USD) (500 Kenya Shillings [KSH]) source of exchange rate: http://www.exchangerate.com/. On 1^st^ November 2008, one USD was equivalent to 81.23 KSH), compared with an average of around 0.37 USD for common, older anti-malarials such as SP and amodiaquine. Malaria diagnosis was predominantly presumptive, based on the presence of fever, in both public and private health facilities [20,21].

### Study design

This study forms part of a cluster-randomized, controlled trial, evaluating the impact of the ACT subsidy intervention on effective malaria treatment of children under five [22]. Data were collected both before (at baseline) and after (at follow-up) the roll out of the intervention and consisted of four main data collection activities: household surveys, provider surveys, mystery-shopper surveys, and focus group discussions. Community randomization of rural sublocations is described in detail elsewhere [22]. Nine sublocations were allocated to the intervention arm, and nine to the control arm, with a buffer zone of two sublocations between selected sublocations. Due to the public information campaign around the subsidized drugs in the intervention arm, blinding was not possible for shopkeepers or data collectors.

### The intervention

The intervention was implemented by the Division of Malaria Control (DOMC) of the Kenyan Ministry of Public Health and Sanitation, Population Services International (PSI), and the Pharmacy and Poisons Board (PPB).

The three main intervention components were provision of subsidized packs of paediatric AL to retail outlets, training of retail outlet staff, and community awareness activities. No interventions were implemented in the control arm. In both intervention and control arms the policy of provision of free AL at government facilities continued unchanged. At baseline, public sector AL stock-outs were common, with only one third of facilities serving the study areas stocking both children’s packs (six- and 12-tablet packs) of AL, but by follow-up this figure had almost doubled to 65% [19; Kangwana, unpublished observation]. In 2006/7 the government had carried out AL awareness campaigns across the country, so both arms had previously received some general information on the current malaria treatment policy.

The intervention targeted retail outlets serving intervention sublocations, which were identified through an outlet census. Outlets were included in the census if they were located in or on the borders of the intervention sublocations and identified by key informants as serving their populations. An initial list of retail outlets was sourced from local public health officers, and updated with input from local chiefs and subchiefs. The list was further amended after visiting the study areas with village elders, through the snowball technique [23] where each shop visited was asked about the presence of other outlets in their area, and by discussions with community members. Enumerated outlets were invited for training if they had been functioning for a minimum period of six months and were identified as selling anti-malarials and/ or antipyretics during the past year. Outlet staff attended a one-day, malaria-related training between August and October 2008 covering clinical diagnosis, treatment, adverse drug reactions, and patient referral. Training materials were developed by the implementation team, building on those used previously for shopkeeper training in Kenya [12].

From November 2008, subsidized AL was provided to trained retail outlets in packs of six tablets (for children aged three to 35 months) and 12 tablets (for children aged 36 to 59 months). The AL was branded as Tibamal, a pretested name derived from the Kiswahili words “*Tiba ya Malaria”* meaning malaria cure, and came with patient instructions suitable for those with low literacy levels. The PPB granted special dispensation for AL to be dispensed over the counter in the intervention arm. PSI sales staff delivered the treatment directly to trained outlets on a monthly basis, at a wholesaler price of 0.10 USD per pack (both packs were the same price). The outlets were instructed to sell the packs at a retail price of 0.25 USD, which was printed on the drug packaging, providing a 150% retailer mark-up, exceeding that of amodiaquine and SP, which generally had retail mark-ups of 50% to 100%.

Trained outlets were supplied with job aids to support dispensing, consisting of a referral flow chart and dosing guidelines. The main community awareness activities began in March 2009, focusing on malaria symptoms, Tibamal availability, and patient adherence. They consisted of community leader workshops, community events; small group discussions and outreach activities. Tibamal was also advertised through posters and paintings on shops selling the treatment, and through distribution of branded headscarves, tee shirts, and pens. A follow-up, supervisory visit was made by the implementation team three months after initial provision of Tibamal supplies to monitor outlet practices.

### Data collection

Data on retailer performance were collected through provider surveys and mystery-shopper surveys, which aimed to determine whether private sector retailers could deliver AL to appropriate standards of quality for the treatment of fever in children under five. The provider survey assessed the anti-malarials stocked, and knowledge and stated practices of the provider related to malaria treatment, such as knowledge of the first-line anti-malarial, malaria symptoms, and advice to provide when dispensing AL. The mystery-shopper survey assessed patient-provider interactions, to provide information on actual rather than self-reported provider practice. Both data collection activities were conducted at baseline (July-August 2008) and follow-up (July-August 2009). The mystery-shopper survey was conducted first, to avoid raising awareness by the shopkeepers of the presence of the survey team. The sampling frame for both surveys was based on the retail census carried out in May 2008 and May 2009, described above. All outlets that had been functioning for at least six months and had sold an anti-malarial or antipyretic within the past year were included in survey data collection.

For the provider survey, written consent was obtained from shopkeepers at the time of the interview. For the mystery-shopper survey, written consent was sought in advance from all shopkeepers interviewed during the retail census. At the time of the census, staff were informed about the nature of the mystery-shopper survey, but details on whether their shop was selected and the date of the survey visit were not revealed. The gap between the census and the mystery-shopper survey was just over a month.

Provider survey data were collected using a pretested structured questionnaire administered to the shopkeeper present at the time of the visit who was most responsible for selling medication. In the mystery-shopper survey, fieldworkers disguised themselves as local residents seeking treatment for a child. All fieldworkers were trained to present the following scenario to the shopkeeper: *a four-year-old child (weighing 15 kg), under their care who has been suffering from a recurring fever for three days, especially at night. The child had no other symptoms, and no medication had been given so far*[13]. The fieldworker discreetly recorded the details of the interaction on a questionnaire away from the outlet, once the interview was complete. All interviews were carried out in each district’s local dialect.

### Data analysis

Based on baseline values of the primary indicator ‘proportion of outlets stocking any unexpired AL’ the study sample size of nine clusters per arm and an average of 26 outlets per cluster was sufficient to detect a 20% point difference at a 0.05 significance level and 80% power [24].

Data were analysed in STATA version 11 (College Station, TX, USA) by a two-stage process, with baseline and follow-up data analysed separately [25]. In the first stage a summary cluster measure was obtained for each cluster. The second stage involved comparing the sets of cluster-specific measures in control and intervention arms at follow-up using the unpaired *t*-test [25]. A crude analysis was carried out on the cluster summaries using the simple two-tailed t-test to obtain the mean percentage difference between the intervention and control arms, 95% confidence intervals (CIs) and standard deviations (SDs) for each outcome. In addition, an adjusted analysis was carried out at follow-up using an individual level logistic regression run on the pooled data set (control and intervention arms). To control for potential confounders the covariates considered were outlet type (specialized drug store or general store), distance of shop to nearest road, whether any staff had clinically related training, and district. All covariates significant at a *p*-value of <0.2 were retained in the regression model. Baseline values for the outcome in question were also included as covariates if a difference of 5% points or more was observed between the arms at baseline. The intervention status of the cluster was not included in the logistic regression model. Rather, the regression model provided the predicted outcome in the absence of the intervention effect. Mean predicted and observed outcomes were obtained per cluster and residuals were obtained by subtracting the predicted outcomes from those observed in each cluster. The *t*-test was used on these residuals to assess the difference in the residuals, which represents the intervention effect adjusted for the covariates included in the logistic regression model. The *t*-test was used for both crude and adjusted analyses, as it has been shown to be highly robust even for small numbers of clusters [25]. For indicators concerning the ‘percentage of outlets providing correct dispensing advice for AL’ in the mystery-shopper survey, the Fisher’s exact test was used to calculate p values, due to the small number of observations in the denominator in the control arm [26]. Distances from retail outlets to nearest roads were calculated using the Euclidean tool in ArcGIS (ESRI, Redlands, CA, USA) spatial analysis tool.

Since the intervention was delivered under operational rather than ideal conditions, outlets in the intervention arm varied in their level of exposure to the intervention. Specifically, not all outlets in the intervention arm were trained, and not all outlets received job aids. The analysis was therefore conducted on both an intention to treat and per-protocol analysis basis. In the intention to treat analysis outlets were analysed in the clusters to which they were randomized, regardless of whether they received the intervention. In the per-protocol analysis only outlets in the intervention cluster that received the intervention were included. Two levels of intensity of intervention were considered: outlets that received the Tibamal training, termed ‘*trained*’, and those that received the Tibamal training and had a Tibamal job aid in the outlet at the time of the interview, termed ‘*trained with job aid’.* No hypothesis testing was carried out to evaluate the statistical significance in differences observed between these subsamples and the population of all outlets in the intervention arm as the groups represent overlapping categories (some outlets were present in two or three of these groups). In both the provider and mystery-shopper surveys, at baseline and also at follow-up, less than 10% of outlets were not interviewed either because the respondent refused to be interviewed or the outlet was closed during visits.

### Ethical approval

Ethical approval was obtained from the Kenya Medical Research Institute (KEMRI) Ethical Review Committee (#1361), the Kenya Pharmacy and Poisons Board Ethical Committee for Clinical Trials (# PPB/ECCT/08/07), and the London School of Hygiene and Tropical Medicine Ethical Review Committee (# 5288). The study is registered with Current Controlled Trials (# ISRCTN59275137).

## Results

### Outlet characteristics

The number of outlets meeting the census selection criteria was 295 in the control arm and 225 in the intervention arm at baseline, and 369 and 351 respectively at follow-up. For the provider survey, a total of 468 outlets were successfully interviewed at baseline and 639 at follow-up; and for the mystery-shopper survey, 499 at baseline and 653 at follow-up (Table 1). The changes in number of outlets surveyed at baseline compared to follow-up were partly due to the fluidity of the market, where over time some shops would close temporarily or permanently, and new ones open. In addition, there is a possibility that fieldworkers became better at identifying outlets at follow-up. In both surveys, general stores constituted over 70% of shops evaluated at baseline and follow-up, with specialized drug shops making up almost all the remainder (Table 1). The mean number of staff serving customers was just under two per outlet at baseline and follow-up. The percentage of outlets with staff with any clinical training, who usually or occasionally served customers, ranged from 15.7% at follow-up in the control arm to 23.5% at baseline in the intervention arm (Table 1). When broken down by outlet type, this averaged 63.6% and 75.7% in specialized drug stores and 9.6% and 4.5% in general stores, across both arms at baseline and follow-up, respectively. Less than 4% of outlets had a child below 16 years of age usually or occasionally serving customers. The mean distance of retail outlets to the nearest road (any road excluding footpaths) was 188 and 327 m in the control and intervention arms respectively at baseline, and 203 and 231 respectively at follow-up (Table 1).

**Table 1:** Characteristics of staff interviewed in the provider survey (cluster summaries from the 9 intervention and 9 control clusters)

	**Baseline**		**Follow up**			
	**Control% (SD) n**	**Intervention% (SD) n**	**Control% (SD) n**	**All Intervention% (SD) n**	**Trained Intervention% (SD) n**	**Trained & Job aid Intervention% (SD) n**
**No. of outlets**	263	205	319	320	154	62
**Outlet type:**						
Specialized drug stores	19.6 (8.4) 49	26.2 (16.3) 53	17.8 (7.3) 56	22.9 (8.7) 74	31.1 (5.5) 49	33.9 (28.6) 21
General stores	80.4 (8.4) 214	73.8 (16.3) 152	81.9 (7.6) 262	77.1 (8.7) 246	68.8 (5.5) 105	66.1 (28.6) 41
Other	0 (0) 0	0 (0) 0	0.3 (1.0) 1	0 (0) 0	0 (0) 0	0 (0) 0
**Outlets with at least one member of staff usually serving customers with the following characteristics:**						
Primary education complete and above	79.2 (9.1) 211	76.8 (17.0) 159	74.8 (11.5) 237	68.7 (13.1) 222	72.6 (18.2) 111	70.4 (23.9) 42
Any clinical training^1^	21.3 (8.1) 55	23.5 (18.8) 51	15.7 (6.7) 50	18.9 (8.4) 63	24.3 (6.3) 40	27.4 (28.2) 16
Below 16 years	3.5 (5.8) 8	3.3 (3.5) 7	3.4 (3.5) 10	2.1 (2.7) 6	2.1 (4.2) 4	2.7 (5.5) 2
**Distance to nearest road:**						
Metres (SD)	187.7 (123.8)	326.6 (286.9)	201.6 (121.5)	231.4 (98.8)	256.3 (156.7)	300.6 (213.6)

n=numerator (denominator for all indicators is shown under No. of outlets).

^1^ Any clinical related training consists of: pharmacy, nurse and medical doctor related training; Pharmacy related training includes pharmacy studied to a certificate or diploma level; Nurse related training includes studying nursing to a certificate level (nurse aid) and diploma level; Medical doctor training includes clinical officer who studied medicine to a diploma level.

Note: In the mystery shopper survey, a total of 499 outlets were successfully interviewed at baseline, 284 and 215 in control and intervention arms respectively, and 653 outlets at follow-up, 336 in the control and 317 in the intervention arm.

Within the subgroup of outlets that had received Tibamal training (*trained*) at follow-up, 31.1% were specialized drug stores, and in outlets that had received training and had a Tibamal job aid (*trained with job aid*) 33.9% were specialized drug stores. Outlet characteristics within the *trained* and *trained with job aid* subgroups were similar to those for all outlets in the intervention arm at follow-up (Table 1), although specialized drug stores were slightly more common among the trained and trained with job aid outlets than among all intervention outlets. The characteristics of outlets and staff surveyed in the mystery-shopper survey were similar to those in the provider survey (data not shown).

### Exposure to the intervention

In the provider survey 71.3% of respondents at baseline and 77.0% at follow-up had heard of AL, across the arms. At follow-up, 13.9% and 91.6% of respondents were aware of Tibamal, in the control and intervention arms, respectively. Also at follow-up, 43.1% of respondents in the intervention arm reported having at least one member of staff that had attended the Tibamal training, compared to only 1.0% in the control arm. Of outlets in the intervention arm, 67 (22.1%) were in possession of a Tibamal job aid and 62 (20.5%) had at least one trained staff member and a Tibamal job aid. No outlet in the control arm had a job aid.

### Anti-malarial availability and storage

During the provider survey, unexpired AL was found in less than 3% of outlets at baseline, across both arms. AL stocking had increased by follow-up to 36.8% in the intervention arm, but remained low at 5.2% in the control arm (Table 2). The difference between the arms at follow-up was significant (adjusted p=0.0001, difference in means: 31.7%; 95%CI: 22.0, 41.3). Tibamal constituted around 95% of all AL available in the intervention arm at follow-up, with no outlets stocking Tibamal in the control arm. Less than 3% of outlets were observed with stocks of any other ACT in both arms and at both time points. At baseline 52.8% of outlets in the control arm stocked a non-ACT anti-malarial (SP, amodiaquine, artemisinin monotherapy, quinine or chloroquine) and 63.7% in the intervention arm. The availability of non-ACT fell by 13 percentage points and 24 percentage points in the control and intervention arms respectively, from baseline to follow-up, though the difference in availability between the arms at follow-up was not significant (adjusted p value=0.5187, difference in means: 0.4%; 95%CI: -7.6, 8.5) (Table 2). The availability of artemisinin monotherapy remained below 5% at baseline and follow-up, and across the arms.

**Table 2:** Availability of anti-malarials (provider survey) (cluster summaries from the 9 intervention and 9 control clusters)

**Availability of anti-malarials:**	**Control% (SD) n/d**	**Intervention% (SD) n/d**	**Difference in means (95%CI)**	**P value Unadjusted *Adjusted***
**Any AL (unexpired):**				
Baseline	0.5 (1.2) 2/263	1.5 (3.2) 3/205		
Follow up	5.2 (4.0) 18/319	36.8 (13.1) 117/320	31.7 (22.0, 41.3)	0.0001 *0.0001*^*1*^
Tibamal trained	-	65.7 (16.1) 103/154		
Tibamal trained & job aid	-	76.8 (32.4) 50/62		
**Tibamal (unexpired):**				
Baseline	-	-		
Follow up	0 (0) 0/319	35.1 (12.3) 111/320	35.1 (26.4, 43.8)	0.0001 *0.0001*^*2*^
Tibamal trained	-	65.3 (15.6) 102/154		
Tibamal trained & job aid	-	76.8 (32.4) 50/62		
**Other ACT:**				
Baseline	0.8 (1.6) 2/263	0 (0) 0/205		
Follow up	2.1 (2.8) 7/319	0.4 (1.1) 1/320	−1.7 (−3.9, 0.4)	0.1058 *0.0580*^*2*^
Tibamal trained	-	0.5 (1.6) 1/154		
Tibamal trained & job aid	-	1.1 (3.3) 1/62		
**Non-ACT therapy:**				
Baseline	52.8 (12.0) 140/263	63.7 (10.4) 130/205		
Follow up	39.6 (10.0) 128/319	40.0 (5.5) 127/320	0.4 (−7.6, 8.5)	0.9082 *0.5187*^*3*^
Tibamal trained	-	54.3 (13.5) 80/154		
Tibamal trained & job aid	-	53.8 (28.1) 35/62		

*n* numerator, *d* denominator, *SD* standard deviation, *CI* confidence interval.

^1^ adjusted for distance to nearest road; ^2^ adjusted for outlet type and district; ^3^ adjusted for outlet type, distance to nearest road and clinical training.

Among the subgroup of *trained* outlets 65.7% stocked AL at follow-up, and among the subgroup of *trained with job aid* outlets, 76.8%, 29 and 40 percentage points higher than among all intervention arm outlets. Availability of non-ACT was also higher in the two subgroups when compared to all outlets in the intervention arm at follow-up. Nine intervention outlets stocked Tibamal even though none of the staff were reported to have attended the training. It was not clear whether this was because the staff member who had attended training was not present on the day of the survey and other staff were not aware they had attended; whether there was some “leakage” of the subsidized drug to untrained shops; or whether the trained staff were no longer working in the outlet.

Outlets stocking AL were assessed to see if the treatment was stored appropriately. The definition of ‘*appropriately*’ was all AL packs kept off the floor, out of direct sunlight, in a dry area and with packaging intact. At follow-up, 78.8% and 81.9% of outlets were observed to be storing all AL stocks appropriately in the control and intervention arms, respectively. The percentages were similar in *trained* outlets and *trained with job aid* outlets and in all intervention outlets. Expired AL stock was found in less than 1% of outlets in both arms and time points.

### Provider knowledge

Data from the provider survey showed that at baseline 37.8% of respondents in the control arm and 34.3% in the intervention arm were able to identify AL as the first-line treatment for uncomplicated malaria. At follow-up, this had improved in both arms, but was significantly greater in the intervention arm compared to the control arm (adjusted p value = 0.0001, difference in means: 24.2%; 95%CI: 14.8, 33.6) (Table 3). Fever, as a symptom of uncomplicated malaria was mentioned by 67.3% of respondents at baseline, across both arms, and increased to 74.3% and 84.0% in the control and intervention arms respectively. Respondents were asked where they would recommend a four year-old child suspected to be suffering from complicated malaria to seek treatment first. Over 70% of respondents correctly said they would refer the child directly to a health facility at baseline, in both arms. This increased to more than 80% at follow-up, across both arms. For all the knowledge indicators described above, respondents from the subgroup of *trained* outlets performed better than the population of all outlets in the intervention arm at follow-up. Outlets that were *trained with job aid* performed better than *trained* outlets with the greatest difference of 10.7 percentage points (82.7% trained: 93.4% trained with job aid) observed in the percentage knowing the first-line anti-malarial (Table 3).

**Table 3:** Provider knowledge (provider survey) (cluster summaries from the 9 intervention and 9 control clusters)

	**Control% (SD) n/d**	**Intervention% (SD) n/d**	**Difference in means (95%CI)**	**P value Unadjusted *Adjusted***
**Percentage knowing the first line anti-malarial for uncomplicated malaria:**				
Baseline	37.8 (9.0) 103/263	34.3 (16.6) 70/205		
Follow up	46.9 (7.9) 151/319	71.1 (10.9) 227/320	24.2 (14.8, 33.6)	0.0001 *0.0001*^*1*^
Tibamal trained	-	82.7 (9.8) 129/154		
Tibamal trained & job aid	-	93.4 (8.2) 58/62		
**Percentage knowing fever as a symptom of uncomplicated malaria:**				
Baseline	66.4 (10.7) 179/263	68.1 (11.6) 142/205		
Follow up	74.3 (8.0) 238/319	84.0 (6.5) 268/320	9.7 (2.4,17.0)	0.0124 *0.0137*^*2*^
Tibamal trained	-	95.2 (7.7) 136/154		
Tibamal trained & job aid	-	96.9 (8.8) 53/62		
**Percentage that would refer a suspected case of complicated malaria in a four year old child to a health facility:**				
Baseline	74.1 (12.0) 194/263	75.9 (7.3) 155/205		
Follow up	83.7 (6.4) 266/319	86.9 (4.0) 278/320	3.2 (−2.1,8.6)	0.2148 *0.0694*^*1*^
Tibamal trained	-	89.6 (7.8) 138/154		
Tibamal trained & job aid	-	90.9 (16.2) 58/62		
**Percentage knowing correct dispensing practices of AL:**				
AL administration				
Baseline	0.7 (1.4) 2/263	0 (0) 0/205		
Follow up	1.2 (1.5) 4/319	13.0 (11.3) 44/320	11.7 (3.7, 19.8)	0.0070 *0.0026*^*3*^
Tibamal trained	-	21.4 (18.3) 36/154		
Tibamal trained & job aid	-	30.7 (22.6) 16/62		
What to do if the child vomits after taking the medication				
Baseline	0 (0) 0/263	0 (0) 0/205		
Follow up	1.5 (2.4) 4/319	9.4 (6.7) 30/320	7.9 (2.9, 12.9)	0.0043 *0.0027*^*4*^
Tibamal trained	-	16.9 (10.8) 27/154		
Tibamal trained & job aid	-	18.2 (18.8) 15/62		
What to do if the child does not get better				
Baseline	46.4 (10.0) 120/263	49.0 (12.6) 99/205		
Follow up	39.8 (13.6) 127/319	66.0 (8.3) 209/320	26.2 (15.0, 37.4)	0.0001 *0.0001*^*4*^
Tibamal trained	-	88.8 (7.1) 136/154		
Tibamal trained & job aid	-	97.9 (4.2) 60/62		
Food to give the child with AL				
Baseline	8.3 (5.7) 22/263	8.6 (9.8) 18/205		
Follow up	6.3 (5.9) 20/319	27.4 (11.2) 86/320	21.1 (12.1, 30.0)	0.0001 *0.0001*^*4*^
Tibamal trained	-	45.1 (5.0) 71/154		
Tibamal trained & job aid	-	61.1 (23.1) 35/62		

*n* numerator, *d* denominator, *SD* standard deviation, *CI* confidence interval.

^1^adjusted for outlet type and district; ^2^adjusted for outlet type; ^3^ adjusted for district and clinical training; ^4^adjusted for outlet type and district.

In the provider survey, respondents were asked about the advice they would give to a caregiver purchasing any brand of AL for their four year-old child. Respondents were asked to advise on AL administration, what to do if the child vomits, what to do if the child does not improve, and foods to administer with the medication (Table 3). Across all the advice points there was significantly higher knowledge (p<0.005) in the intervention arm, compared to the control arm, at follow-up. The greatest difference was observed in advice to give ‘if the child does not get better’; at baseline 47.7% of respondents knew the correct advice, and at follow-up 39.8% and 66.0% in the control and intervention arms respectively (adjusted p value=0.0001; difference in means: 26.2%; 95%CI: 15.0, 37.4). The least improvement was observed in ‘what to do if the child vomits after taking the medication’, where no respondents knew the correct response at baseline, and at follow-up this increased to only 1.5% and 9.4% in the control and intervention arms respectively (adjusted p value: 0.0027; difference in means 7.9%; 95%CI: 2.9, 12.9). Across these advice indicators, the subgroup of *trained* outlets performed better than the population of all outlets in the intervention arm, with the greatest difference observed in ‘what to do if the child does not get better’ where there was a 22.8 percentage point difference (66.0% all outlets; 88.8% trained outlets). The least improvement was observed in ‘what to do if the child vomits’ where there was a 7.5 percentage point difference (9.4% all outlets; 16.9% trained outlets). Trained outlets with job aids performed better than trained outlets, with the greatest difference in the correct advice on foods to give the child (45.1% trained outlets; 61.1% trained with job aid), and the least difference in what to do if the child vomits (16.9% trained outlets; 18.2% trained outlets with job aids) (Table 3).

### Provider behaviour

At baseline anti-malarials were dispensed to 25.2% of mystery shoppers in the control arm and 40.7% in the intervention arm, and this was little changed at endline, with 20.2% in the control arm and 40.3% in the intervention arm (Table 4). This remained fairly similar at follow-up. The reasons for the higher frequency of anti-malarial dispensing in the intervention arm at both time points were not clear. Less than 1% of mystery shoppers were sold AL at baseline, across both arms. At follow-up, sales of AL remained low at 1.8% in the control arm, but were significantly higher at 25.4% in the intervention arm (adjusted p=<0.0001; difference in means: 23.6%; 95%CI: 18.7, 28.6). If only those intervention outlets are considered which had a staff member who had attended Tibamal training, and had Tibamal in stock during the provider survey, 60% dispensed Tibamal to the mystery shoppers. Of the 40% (39 outlets) that did not dispense Tibamal, 16 (mainly general stores) referred the mystery shopper to a specialized drug store, 13 (mainly drug stores) dispensed another anti-malarial (mainly SP), 6 dispensed an antipyretic only, 3 referred to a general shop, and 1 referred to a health facility.

**Table 4:** Provider practices (mystery shopper survey) (cluster summaries from the 9 intervention and 9 control clusters)

	**Control% (SD) n/d**	**Intervention% (SD) n/d**	**Difference in means (95%CI)**	**P value Unadjusted *Adjusted***
**Percentage of outlets dispensing any anti-malarial:**				
Baseline	25.2 (8.9) (74/284)	40.7 (9.3) (88/215)		
Follow up	20.2 (3.7) (68/336)	40.3 (6.5) (127/317)	20.1 (14.8, 25.4)	0.0001 *0.0260*^*1*^
Tibamal trained		56.6 (23.0) (88/144)		
Tibamal trained & job aid		70.8 (30.0) (37/58)		
**Percentage of outlets dispensing any AL:**				
Baseline	0.5 (1.6) (2/284)	0.0 (0) (0/215)		
Follow up	1.8 (1.3) (6/336)	25.4 (6.9) (77/317)	23.6 (18.7, 28.6)	0.0001 *0.0001*^*2*^
Tibamal trained		43.3 (18.9) (67/144)		
Tibamal trained & job aid		62.7 (31.0) (31/58)		
**Percentage of outlets asking about at least one danger sign:**				
Baseline	26.5 (14.1) (21/284)	21.6 (12.4) (14/215)		
Follow up	27.4 (10.7) (90/336)	33.3 (14.2) (97/317)	4.9 (−7.9, 17.7)	0.4295 *0.2667*^*3*^
Tibamal trained		46.3 (25.5) (58/144)		
Tibamal trained & job aid		51.3 (30.0) (28/58)		
**Percentage of outlets providing the correct dispensing advice for AL:**				
AL administration				
Baseline	0.0 (0) (0/2)	0.0 (0) (0/0)		
Follow up	0.0 (0) (0/6)	31.3 (16.3) (24/77)	31.3 (16.8, 45.8)	0.175
Tibamal trained		34.2 (18.0) (23/67)		
Tibamal trained & job aid		45.2 (30.5) (12/31)		
What to do if the child vomits after taking the medication				
Baseline	0.0 (0) (0/2)	0.0 (0) (0/0)		
Follow up	0.0 (0) (0/6)	2.5 (3.9) (2/77)	2.5 (−1.9, 6.8)	1.000
Tibamal trained		2.8 (5.7) (2/67)		
Tibamal trained & job aid		4.4 (8.8) (2/31)		
What to do if the child does not get better				
Baseline	0.5 (1.6) (1/2)	0.0 (0) (0/0)		
Follow up	0.0 (0) (0/6)	35.3 (13.1) (26/77)	35.3 (23.6, 47.1)	0.170
Tibamal trained		37.7 (12.6) (24/67)		
Tibamal trained & job aid		38.9 (24.1) (14/31)		
Food to give the child with AL				
Baseline	0.0 (0) (0/2)	0.0 (0) (0/0)		
Follow up	0.0 (0) (0/6)	13.7 (14.4) (9/77)	13.7 (0.8, 26.6)	1.000
Tibamal trained		13.3 (14.2) (8/67)		
Tibamal trained & job aid		11.9 (14.8) (5/31)		

^1^adjusted for outlet type and clinical training; ^2^adjusted for outlet type; ^3^adjusted for district and outlet type.

*n* numerator, *d* denominator, *SD* standard deviation, *CI* confidence interval.

Note: In the mystery shopper survey, a total of 499 outlets were successfully interviewed at baseline, 284 and 215 in control and intervention arms respectively, and 653 outlets at follow-up, 336 in the control and 317 in the intervention arm.

An average of 24.5% of respondents across both arms asked their clients whether the child had at least one danger sign at baseline. This improved slightly in the intervention arm at follow-up, however the difference between the arms was not statistically significant (adjusted p value=0.2667; difference in means 4.9%; 95% CI: -7.9, 17.7). For both of these provider-behaviour indicators *trained* outlets performed better than all outlets in the intervention arm, with the greatest difference observed in outlets dispensing AL (25.4% all outlets; 43.3% trained outlets). Similarly, *trained outlets with job aids* performed better than *trained* outlets, with the greatest difference also observed in dispensing of AL (43.3% trained outlets; 62.7% trained with job aid).

Less than 1% of providers gave correct dispensing advice for AL on any of the counselling points at baseline, across both arms. At follow-up there were improvements in advice in the intervention arm, but the differences between the arms were not significant for any counselling indicators related to AL dispensing. The greatest difference was observed in what to do if the child does not get better, which remained 0% in the control arm compared with 35.3% in the intervention arm (difference in means: 35.3%; 95% CI: 23.6, 47.1). *Trained* outlets performed better than all outlets in the intervention arm on advice on administration of AL, and what to do if the child does not get better. Little difference was observed between the groups in what to do if the child vomits, and foods to give the child. *Trained outlets with job aids* performed better than *trained* outlets in all counselling points apart from on advice on foods to give the child.

### Cost to consumers of artemether-lumefantrine

In the mystery-shopper survey, at baseline there were only two doses of AL sold, at a cost of 2.46 and 2.22 USD. At follow-up, the 12 tab Tibamal was sold at a median price of 0.25 USD (IQR: 20–20), which was the recommended retail price. Of those not paying the recommended price, two paid 0.37 USD, another two paid 0.49 USD because of buying two packs of the six-tab to meet the required dose, and one paid 0.74 USD.

## Discussion

Before discussing the results in more detail a number of limitations in the provider and mystery-shopper surveys should be highlighted. Shops that had undergone Tibamal training were identified by asking the respondent if any of the staff had attended the training, but it is possible that some responses were inaccurate due to recall bias or lack of awareness of training of fellow staff members. In addition, given the prior consent process, it is also possible that retailers were suspicious and therefore altered their practice and the advice they gave to the mystery shopper. However, if this were the case then the data would display ‘best practice’ of providers, while still showing considerable room for improvement, especially in areas such as the provision of appropriate counselling. In the scenario, the mystery shoppers waited for the retailer to recommend treatment and paid whatever price they were asked to. Other data (Kangwana and colleagues, unpublished observations from Tibamal focus group discussions and provider survey reports) suggest that in practice the consumer often asks for a specific treatment instead of the provider recommending it, so real-life interactions may be somewhat different. It is also possible that there was some contamination of the control arm outlets, which could have heard some of the communication activities. However, results indicated that such exposure was low, with only 1% of control arm respondents saying that they had attended the Tibamal training, 14% having heard of Tibamal, and no outlets stocking Tibamal.

The intervention was able to significantly improve the percentage of outlets stocking AL, and more than 90% of the AL available at follow-up in the intervention arm was Tibamal. This indicates a willingness of shopkeepers to take part in the intervention and make the treatment available in their outlets. The intervention had an effect on most provider knowledge indicators. Significantly more providers in the intervention arm compared to the control arm knew AL was the first-line treatment for uncomplicated malaria and knew fever as a symptom of uncomplicated malaria. Providers were also more knowledgeable on correct dispensing practices for AL, than those in the control arm. However, although the difference observed between the arms was significant, there remains a need for renewed effort to improve these components of knowledge and prescription. For example, for the scenario of ‘what to do if the child does not get better’, only 66% of providers could give the correct response. In addition, findings from the mystery-shopper survey revealed that knowledge was not always transferred into practice, since no significant improvements were observed in any of the four appropriate counselling indicators.

The mystery-shopper survey revealed that the intervention not only encouraged shopkeepers to stock AL but also significantly encouraged them to dispense AL to clinically diagnosed cases of uncomplicated malaria. This is in line with findings from the household surveys, which showed that a significantly greater percentage of febrile children in the intervention arm were treated with AL compared to the control arm at follow-up [22]. The findings in this paper on changes in provider behaviour also serve to strengthen the claim that the changes observed in the household survey were very likely due to the Tibamal intervention. Encouragingly, the mystery shopper data showed providers adhering to the recommended retail price of Tibamal. Findings from the household survey also revealed that >95% of caregivers purchased Tibamal at its recommended retail price [22]. Printing of the recommended price on Tibamal packaging as well as making consumers aware of the subsidy through the community activities may have contributed to providers not inflating Tibamal prices. Also, the way in which the pricing was structured meant that even with the subsidy, the retailer mark-up would be greater than for other more commonly prescribed anti-malarials, which may have facilitated both stocking of Tibamal by retailers and appropriate pricing.

The intervention did not significantly increase the percentage of providers that would refer complicated cases of malaria directly to a health facility. The data show that even without the intervention, around 80% of shopkeepers stated that they would automatically refer these cases to a health facility, but since immediate specialized care is required for complicated cases, one would hope to see a referral rate of close to 100%. The intervention was also not able to significantly increase the percentage of providers asking for at least one danger sign, an important way of identifying severe cases. It could be that providers instead tended to rely on the consumer to provide all the necessary information without being probed. Identifying and implementing ways to improve enquiries about danger signs is therefore important, in addition to providing caregivers with the knowledge of when to bypass the retail sector and go straight to a health facility. Although the intervention improved the share of AL among mystery-shopper purchases it had no significant impact on decreasing the availability of non-ACT in outlets. There was a decline in non-ACT availability at follow-up, but this was observed in both arms and was thought to be as a result of a government directive to halt the production and supply of less effective monotherapy at the time of the survey. Availability of artemisinin monotherapy was not a concern, with less than 5% of outlets stocking this treatment, probably due to low demand as a result of its high cost compared to other anti-malarial monotherapy [27].

Overall, it seemed that the greater exposure shopkeepers had to all components in the intervention, the better they tended to perform. Outlets that had received training seemed to perform better than all outlets in the intervention arm, and outlets that had received both training and job aids seemed to perform better than outlets just exposed to training. This shows the importance of ensuring that implementation of the intervention is as ‘ideal’ as possible [28]; had higher coverage of training and job aids been achieved, even more substantial improvements in provider behaviour and treatment coverage would likely have been achieved.

These findings are of particular importance given the current roll out of similar ACT subsidies under the Affordable Medicines Facility - malaria (AMFm) on a national scale in Kenya and seven other countries, also accompanied by training and communications activities [29]. In Kenya AMFm training of retail providers was limited to registered pharmacists who were the only retailers officially allowed to stock the AMFm subsidized product, although in practice it was widely available in unregistered pharmacies [30]. By demonstrating variation in performance in relation to intervention intensity, and highlighting areas of particularly weak provision, these results can be used to identify potential strategies to enhance provision of subsidized ACT under AMFm and other similar subsidy mechanisms.

The authors of this paper are not aware of any other studies that explore the effect of an intervention including an ACT subsidy on the performance of private sector retailers in the treatment of presumptive malaria. Several studies in sub-Saharan Africa have however evaluated the effect of other interventions to improve the quality of care received from the retail sector in the treatment of presumptive malaria, mostly with anti-malarial monotherapy. The majority of the interventions included training of either providers or users, and combined this with other supporting activities such as provision of job aids, follow-up monitoring and provision of prepackaged anti-malarial treatment with pictorials to guide administration. The outcomes of the interventions varied between studies but the majority showed positive outcomes. Overall the interventions were able to improve provider knowledge on signs and symptoms of malaria [31,32], and the proportion of providers giving correct treatment and dose [14,33]. Some interventions increased provision of correct advice on administration [12,31], and asking for danger signs [31]. Interventions were also able to improve the availability of anti-malarials in outlets [15,31]. Studies differed in their design, data analysis techniques used and the type of outcome measures used. Few studies carried out hypothesis testing on their outcome results so it is difficult to interpret the importance of any observed differences, and some studies had limitations, for example very small sample sizes or no appropriate comparison group. All these factors make it difficult to make a direct comparison of outcomes in these studies with the data presented here.

Exploration of the context in which the survey took place, together with interviews with participants in the intervention through focus group discussions (Kedenge and colleagues, unpublished observations from the Tibamal focus group discussion report), have provided some insight into the findings and how the results could have been further improved. At follow-up, only 43% of outlets in the intervention arm were identified as trained. Several possible reasons were given including that some businesses trained at baseline had closed due to lack of capital; some businesses relocated to other areas or had changed their type of business, for example, from a general store to a bicycle spare part shop. Also, recently opened but untrained shops had not had the opportunity to be trained. This highlights the need to hold regular courses to ensure that a well-trained cadre of shopkeepers is maintained. In addition some providers thought that the training should have been longer, refresher courses should have been included and the training could have better catered for illiterate shopkeepers. Furthermore in response to the low percentage of patients being counselled, some shopkeepers were said to work in a very busy environment, having to attend to more than one customer at a time, making it difficult to discuss these issues with caregivers in any detail. As this is unlikely to change, this highlights the merits of also providing this information directly to consumers through communication activities.

Care should be taken when extrapolating or generalising the findings of this pilot to other areas. Since Tibamal was being supplied directly to outlets, there is the possibility that monthly contact with PSI staff distributing AL may have had the effect of influencing providers to work at their best. Other factors affecting the generalizability of these findings have been discussed by Kangwana *et al.*[22].

Finally, the World Health Organization now recommends that all suspected cases of malaria should be parasitologically diagnosed before treatment, where diagnostics are available [1]. Therefore further research is required into understanding how diagnostics will change provider practices, and assessing strategies to ensure diagnostics are used appropriately and supplied at an affordable price.

## Conclusion

The private sector remains a preferred source of anti-malarial treatment in much of sub-Saharan Africa, including Kenya. It is, therefore, important to support and enable providers to provide appropriate and rational management of malaria. This study has shown how an intervention comprising of subsidized pre-packaged AL, retailer training and community awareness activities can improve certain aspects of provider knowledge and practices of presumptive malaria treatment.

The intervention significantly increased provider awareness of the government’s recommended first-line treatment for uncomplicated malaria, and along with granting of over-the-counter status to AL, significantly increased its availability. The intervention also significantly increased the percentage of providers recommending and dispensing AL, and encouraged providers to pass on the subsidy to customers, making the treatment more affordable. Further research is needed to improve aspects of care where the intervention had minimal impact, particularly provider knowledge and behaviour around danger signs of malaria, and counselling practices.

## Competing interests

The authors declare that they have no competing interests.

## Authors’ contributions

BPK, AJN, GWF, RWS and CAG designed the study; BPK, AMN, AJN, GWF, JET and CAG analysed the data; BPK and SVK collected the data; BPK enrolled participants; BPK wrote the first draft of the paper; BPK, SVK, AMN, VAA, AJN, JP, GWF, JET, RWS and CAG contributed to the writing of the paper; JP ensured that regulatory requirements for pharmacovigilance were met; JET advised on the interpretation of the study results; RWS reviewed all primary and secondary analysis; BPK, SVK, AMN, VAA, AJN, JP, GWF, JET, RWS and CAG agreed with the manuscript’s results and conclusions. All authors read and approved the final manuscript.
